# Supplementary material for: Modelling hCDKL5 Heterologous Expression in Bacteria
Source: Metabolites. 2021 Jul 28;11(8):491. doi: 10.3390/metabo11080491 (PMC8401935; doi:10.3390/metabo11080491)

Corresponding Author:

Prof. M. Luisa Tutino,

Dipartimento di Scienze Chimiche, Università di Napoli "Federico II", Complesso universitario Monte Sant'Angelo, Via Cynthia, 80126 Napoli, Italia.

Phone: +39-081674317,

Fax: +39-081-674313,

E-mail:tutino@unina.it

Running Head: Soluble *h*CDKL5 production in *P. haloplanktis* TAC125 at 0 °C

Soluble recombinant protein production in *Pseudoalternomonas haloplanktis* TAC125: the case study of the full-length human CDKL5 protein.

Marzia Calvanese[#], Andrea Colarusso[#], Concetta Lauro[#], Ermenegilda Parrilli and Maria Luisa Tutino*.

Department of Chemical Sciences – University of Naples “Federico II” –Via Cintia, 4 80126 Naples – Italy

[#] these authors contributed equally to this work

* Corresponding author

Abstract

The Antarctic bacterium *Pseudoalteromonas haloplanktis* TAC125 is an unconventional protein production host displaying a notable proficiency in the soluble production of difficult proteins, especially of human origin. Furthermore, the accumulation of recombinant products in insoluble aggregates has never been observed in this bacterium, indicating that its cellular physicochemical conditions and/or folding processes are rather different from those observed in mesophilic bacteria. The ability of this cell factory was challenged by producing a human protein, the cyclin dependent kinase-like 5 (*h*CDKL5) in the bacterium cytoplasm at 0 °C. Human CDKL5 is a serine/threonine protein kinase characterised by the absence of a defined structure for the last two/third of its sequence, one of the largest intrinsically disordered regions so far observed in a human protein. This large unstructured domain makes difficult its production in most of the conventional hosts since the recombinant product accumulates as insoluble aggregates and/or is heavily proteolyzed. As the full-length *h*CDKL5 production is of great interest both for basic science and as protein drug for an Enzyme Replacement Therapy, its production in the Antarctic bacterium was tested by combining the use of a regulated psychrophilic gene expression system with the use of a defined growth medium optimised for the host growth at sub-zero temperature. This is the first report of soluble and full-length recombinant production of *h*CDKL5 protein in a bacterium.

Key Words: *Pseudoalteromonas haloplanktis* TAC125, human CDKL5_5, psychrophilic gene expression system pMAV, GG medium, intrinsically disordered proteins.

1. Introduction

Pseudoalteromonas haloplanktis TAC125, a psychrophilic Gram-negative bacterium isolated from Antarctic seawater (1), is considered one of the most interesting unconventional hosts for the recombinant production of difficult-to-express proteins, thanks to favourable physiological features, including fast growth at low temperatures and efficient protein synthesis (1, 2). Furthermore, a series of proof-of-concept studies have already demonstrated its feasibility for the recombinant expression of human proteins (3, 4, 5). During the last decade, the number of reliable genetic systems for the recombinant gene expression in *P. haloplanktis* TAC125 was significantly increased, and the recently reported possibility to produce proteins within a range of temperature from 15 to −2.5 °C enhances the chances to improve the conformational quality and solubility of recombinant proteins (6). Moreover, the development of synthetic media and of a finely regulated gene-expression system inducible by D-galactose (6) allowed for the production of a recombinant protein at sub-zero temperature for the first time, thus providing an innovative strategy for the recombinant production of "difficult" proteins.

In the present chapter we describe the main issues encountered and the implemented strategies towards the successful production of a human protein by the psychrophilic cell factory using the regulated gene expression system inducible by D-galactose (pMAV) and the synthetic medium GG (6).

The study' object is the human cyclin-dependent kinase-like 5 (CDKL5), a serine/threonine protein kinase almost ubiquitous in vertebrates and very conserved over evolution. In mammals it is produced mainly in the brain and in some other tissues, and has been reported to be essential for the normal development of the central nervous system. Mutations in its gene are known to cause a severe neurodevelopmental disorder (CDKL5 deficiency disorder) (7, 8) accompanied by intractable epilepsy.

This protein is involved in different molecular processes and many aspects of its basic biology are still not completely understood, but it is known that it exerts its action both in the cell nucleus, in the cytoplasm and at the level of the cytoplasmic membrane. Such heterogeneity in terms of cellular localization and function is a common feature of Intrinsically Disordered Proteins (IDPs) (9), which are characterised by uncommon flexibility allowing them to meet several different molecular partners. Human CDKL5 responds to these structural criteria, as only about one-third of the protein (the N-terminal one) is precisely structured, while the remaining part is predicted to be flexible (10). This structural peculiarity makes human CDKL5 a difficult-to-express protein in the most conventional hosts. It tends to be either heavily proteolyzed or to accumulate in insoluble aggregates. As observed in previous reports, when CDKL5 was expressed in *E. coli* the recombinant protein was exclusively obtained in the insoluble fraction (11, 12).

Human CDKL5 is known to exist in five isoforms resulting from alternative splicing (13, 14). In this chapter, the recombinant production of the isoform *h*CDKL5_5 (previously known as CDKL5 115) is reported (15).

In detail, we describe the procedure for the cloning of *hCDKL5* gene into the pMAV psychrophilic gene expression system and its mobilization into *P. haloplanktis* TAC125 cells. The recombinant Antarctic strain was then grown in optimised culture conditions in the synthetic medium (GG) (6) at 0 °C, and different induction conditions were explored to produce the human protein. The recombinant expression was followed by monitoring *h*CDKL5_5 biosynthesis to define optimal process conditions to produce the human enzyme in soluble form.

2. Materials

2.1. Bacterial strains

1. *Pseudoalteromonas haloplanktis* TAC125. This strain was kindly provided by C. Gerday, University of Liege, Belgium. The strain was isolated from the seawater in the surroundings of the Dumont d'Urville Antarctic station (66°40' S, 40°01' E) during the 1988 summer campaign of the «Expeditions Polaires Française» in Terre Adélie (1).

2. *E. coli* DH5α (*supE44, ΔlacU169* (ϕ80 *lacZΔM15*) *hsdR17, recA1, endA1, gyrA96, thi-1, relA1*). This strain was used as the host for the gene cloning.

3. *E. coli* strain S17-1(λ*pir*) (*thi, pro, hsd* (r^- *m+) recA::RP4-2-TCr::Mu Kmr::Tn7 Tpr Smr* λ*pir*). This strain was used as the donor strain in intergeneric conjugation experiments (16).

4. Bacteria can be stored indefinitely in cultures containing 20 % (v/v) of sterile glycerol (sterilize by autoclaving for 20 minutes at 1 atm on liquid cycle) at low temperature (from -20 to -70 °C). Add 0.25 mL of 80% (v/v) glycerol to 0.75 mL of bacterial culture, mix gently and freeze rapidly placing the tube in a dry ice/acetone mix.

2.2. Solutions

1. 100 mg/mL ampicillin stock solution: dissolve 1 g of ampicillin powder in 8 mL of deionised water. Adjust the volume of the solution to 10 mL with dH_2O and sterilize by filtration through a 0.22 μm sterile filter. Split the obtained stock solution in 10 aliquots of 1 mL each in sterile polypropylene tubes and store them at -20 °C.

2. 1x TAE buffer for agarose gel electrophoresis: 40 mM Tris-acetate, 1 mM EDTA (pH 8.0). Make a 50x TAE stock solution by mixing 242 g of Tris base, 57.1 mL of glacial

acetic acid, 100 mL of 0.5 M EDTA (pH 8.0) and adjust the volume of the solution to 1 litre with dH_2O. Store at room temperature (RT) up to 1 year.

3. 0.5 M EDTA, pH 8.0: dissolve 186.1 g of EDTA in 800 mL of dH_2O. Adjust the pH to 8.0 with NaOH (about 20 g of NaOH pellets) and adjust the volume of the solution to 1 L with dH_2O.

4. 3 M NaCl stock solution: Dissolve 87.6 g of NaCl in 500 mL of dH_2O.

5. GelRed precast agarose gel. BiotiumGelRed 10,000x solution is diluted to 1x by adding 3.5 µL of the stock solution to 35 mL of molten agarose gel. Once casted, protect the gel from light with aluminium foil (*see* **Note 1**).

6. 1 M D-galactose stock solution: Dissolve 1.8 g of solid D-galactose in 8 mL of dH_2O. Adjust the volume of the solution to 10 mL with dH_2O and sterilize by filtration through a 0.22 µm sterile filter. Store at -20°C up to 1 year.

7. 1 M DTT stock solution: Dissolve 3.09 g of DTT in 20 mL of dH_2O. Sterilize by filtration through a 0.22 µm sterile filter. Dispense into 1 mL aliquots and close them under nitrogen flow. Store the aliquots at -20°C.

8. 4x SDS-PAGE loading buffer: 250 mM Tris-HCl pH 6.8, 40% (v/v) glycerol, 4% (w/v) SDS, 200 mM DTT and 0.02% bromophenol blue. This buffer lacking DTT can be stored at RT. DTT should be added just before the use from a 1 M stock solution.

9. 0.5 M Tris-HCl (pH 6.8): Dissolve 60.55 g of Tris base in 800 mL of dH_2O. Adjust the pH to 6.8 with HCl and add dH_2O to make up a final volume of 1 L.

10. 0.5% (w/v) bromophenol blue: Dissolve 0.25 g bromophenol blue powder in 45 mL of dH_2O. Shake well to dissolve the dye and then adjust the volume of the solution to 50 mL with dH_2O. Store at RT.

11. 5x Running buffer: Dissolve 15.1 g of Tris base, 94 g of glycine, and 5 g of SDS in 900 mL of dH_2O. Adjust the volume of the solution to 1 L with dH_2O.

12. 0.5 M Sodium Phosphate buffer, pH 7.8: Dissolve 68.9 g of NaH_2PO_4 in 900 mL of dH_2O. Adjust the pH to 7.8 with NaOH and add dH_2O to make up a final volume of 1 L.

13. Lysis buffer: mix 20 mL of 0.5 M Sodium Phosphate buffer, pH 7.8 with 16.67 mL of 3M NaCl, 0.4 mL of 0.5M EDTA, pH 8.0, 0.1 mL of 1M DTT solution, and 1 mL of Triton X-100. Add dH_2O to 90 mL, and eventually adjust the pH to 7.8. Take the final solution volume to 100 mL with deionized water.

14. Western blot 1x Transfer buffer: Dissolve 3.03 g Tris base, 14.41 g glycine in 800 mL of dH_2O. Add 200 mL methanol. Adjust the volume of the solution to 1 L with dH_2O.

15. Western blot Blocking buffer: Dissolve 50 g Skimmed Milk in 1 L of PBS buffer (5% w/v). Add 0.5 mL Triton X-100 (0.05 % v/v) and mix.

16. Western blot Washing buffer: Add 0.5 mL of Triton X-100 in 1 L of PBS buffer (0.05 % v/v) and mix.

17. 1x PBS: Dissolve 8 g NaCl, 0.2 g KCl, 1.44 g Na_2HPO4 and 0.24 g KH_2PO_4 in 800 mL of dH_2O. Adjust the pH to 7.4 with HCl. Add dH_2O to 1 L. Sterilize by autoclave.

18. Schatz Salts stock solutions:

a. 100x KH_2PO_4 (100 g/L): dissolve 100 g of KH_2PO_4 in 800 mL of dH_2O. Adjust the pH to 7.0 by NaOH addition. Add dH_2O to 1 L. Sterilize by filtration through a 0.22 µm sterile filter. Store at RT up to 6 months.

b. 300x $MgSO_4*7H_2O$ (60 g/L): dissolve 60 g of $MgSO_4*7H_2O$ in 800 mL of dH_2O. Add dH_2O to 1 L. Sterilize by filtration through a 0.22 µm sterile filter. Store at RT up to 6 months.

c. 500x $FeSO_4*7H_2O$ (2.5 g/L): dissolve 2.5 g of $FeSO_4*7H_2O$ in 800 mL of dH_2O. Adjust the pH to 4.0-5.0 by NaOH addition. Add dH_2O to 1 L. Sterilize by filtration through a 0.22 µm sterile filter. Store at RT up to 6 months.

d. 3,000x $CaCl_2*2H_2O$ (15 g/L): dissolve 15 g of $CaCl_2*2H_2O$ in 800 mL of dH_2O. Adjust the pH to 7 by NaOH addition. Add dH_2O to 1 L. Sterilize by filtration through a 0.22 µm sterile filter. Store at RT up to 6 months.

2.3. Media

1. LB medium (1L) (17): 10 g Bacto-Tryptone, 5 g Bacto-Yeast Extract, 10 g NaCl. Add 950 mL of dH_2O. Shake until the solutes have dissolved. Adjust the volume of the solution to 1 L with dH_2O. Sterilize by autoclaving for 20 min at 1 atm on liquid cycle. Let it cool down and store at RT. When required, add 1 mL of sterile ampicillin stock solution. To prepare solid medium, add 15 g/L Bacto-Agar just before autoclaving.
2. TYP Medium (1L) (18)): 16 g Bacto-Tryptone, 16 g Bacto-Yeast Extract, 10 g NaCl, add 950 mL of dH_2O. Shake until the solutes have dissolved. Adjust the volume of the solution to 1 L with dH_2O. Sterilize by autoclaving for 20 min at 1 atm on liquid cycle. When required, add 1 mL of sterile ampicillin stock solution. To prepare solid medium, add 15 g/L Bacto-agar just before autoclaving.
3. GG medium (1L) (6): 10 g L-glutamic acid monosodium salt monohydrate, 10 g gluconic acid sodium salt, 10 g NaCl, 1 g NH_4NO_3 in 900 mL of dH_2O. Adjust the pH to 7.8 with NaOH and reach 1 L before autoclaving. After sterilization, dilute Schatz Salts stock solutions to 1x concentration (*see* **Note 2**).
When required, add 1 mL of sterile ampicillin stock solution to 1L medium to have a 100 µg/mL final concentration. For bacterial growths at 0 °C a lower amount of antibiotic was used, i.e. 0.25 mL per Litre of medium (25 µg/mL final concentration).

2.4. Reagents

1. Restriction enzymes: *Nde*I, *Xho*I, *Sal*I

2. Calf Intestinal alkaline Phosphatase, CIP

3. T4 DNA Ligase

4. QIAquick® PCR Purification kit

5. QIAprep® Spin Miniprep kit

6. Anti-CDKL5 Antibody (D-12, Santa Cruz Biotechnology)

7. Peroxidase conjugated anti-mouse IgG

8. Clarity Western ECL substrate

9. cOmplete mini Protease Inhibitor cocktail tablet

2.5. Vectors

1. pMAV: a psychrophilic gene expression system in which the expression is induced by galactose supply (*see* **note 3**).

2. pET28a-*hCDKL5*: an expression vector containing the *E. coli* codon-optimized sequence coding for the human CDKL5 isoform 5 (*see* **note 4**).

3. Methods

3.1. *h*CKDL5_5 expression vector construction

HCDKL5_5 gene was extracted from pET28a-*hCDKL5* vector by double hydrolysis with *Nde*I and *Xho*I restriction enzymes and cloned into pMAV plasmid double digested with

*Nde*I/*Sal*I. *Xho*I and *Sal*I produced complementary cohesive ends and this allowed for a direct ligation reaction without further modifications.

1. Digest the pET28a-*hCDKL5_5* vector with *Nde*I and *Xho*I enzymes. Restriction hydrolysis is performed by using 5 enzyme Units/μg of DNA, in the reaction conditions defined by the manufacturer.

2. The DNA is purified after agarose gel electrophoresis using a commercial purification kit.

3. The pMAV vector is digested with *Nde*I and *Sal*I enzymes.

4. The DNA is purified using a commercial purification kit.

5. The 5' phosphate groups of the cleaved vector are dephosphorylated by treatment with calf intestinal alkaline phosphatase (CIP) (1 U/pmol of 5' phosphate ends) for 30 min at 37°C by using the appropriate buffer delivered with the enzyme.

6. The dephosphorylated DNA is then purified using a purification kit following the manufacturer's instructions.

7. The cleaved dephosphorylated vector is then ligated to the digested fragment by using ligation reactions, by the means of T4 DNA ligase according to the supplier's instructions.

8. The ligation reaction mixture is used directly for the transformation of the chemically competent bacteria (*E. coli* DH5α strain).

9. Recombinant clones are selected on LB agar plates containing 100 μg/mL ampicillin as the selection agent.

10. Plasmids are isolated from ampicillin resistant clones by a suitable mini prep kit, and the presence of the appropriate insert is verified by restriction digestion analysis.

3.2. Construction of *P. haloplanktis*TAC125 (pMAV-*hCDKL5_5*) recombinant strain

1. The resulting vector pMAV-*hCDKL5_5* is mobilised into *P. haloplanktis* TAC125 by intergeneric conjugation (16). After the mating step, cells are suitably diluted and plated on TYP solid medium containing 100 μg/mL ampicillin and incubated at 4 °C to select recombinant *P. haloplanktis* TAC125 (the low temperature hinders *E. coli* growth as colonies on solid media).

2. 3 isolated colonies are picked and each one inoculated in 3 mL of TYP liquid medium containing 100 μg/mL ampicillin and incubated at 4°C under shaking (250 rpm) for 48-72 h.

3. Plasmidic DNA is extracted from each clone by using a miniprep kit and recombinant plasmid clones are screened by restriction digestion analysis.

3.3. *h*CDKL5-5 production

All psychrophiles, including *P. haloplanktis* TAC125, are exposed to permanent oxidative stress at low temperatures, which originates from increased oxygen solubility. Therefore, *h*CDKL5_5 production trials were performed at different liquid-to-gas ratios in 1 L shaken flasks. Recombinant *P. haloplanktis* (pMAV- *hCDKL5_5*) batch cultivation was performed in different conditions:

- 200 mL of GG supplemented with Schatz Salts (20% fill volume)
- 350 mL of GG supplemented with Schatz Salts (35% fill volume)
- 700 mL of GG supplemented with Schatz Salts (70% fill volume)

In all the above conditions 25 µg/mL ampicillin was added to maintain a suitable selection for the growth of recombinant clones. All the production trials were performed in 1 L Erlenmeyer flasks at 0 °C with shaking at 160 rpm in Nüve-Cooled incubator (model ES120). 40 mM of D-galactose was used as the inducer of recombinant expression and the production was evaluated after 8, 24 and 48 h post induction. Cell growth was monitored by measuring the optical density (OD) at 600 nm using a spectrophotometer.

3.3.1. Pre-culture

The viability of the pre-cultured cells is crucial for a satisfying process outcome. At the time of the inoculation of the production batch, the bacteria must be in middle exponential phase in the same medium that will be used for the expression growth. Furthermore, temperature control is crucial for the reproducibility of the experiments (*see* **Note 5**).

1. From a glycerol stock streak the *P. haloplanktis* TAC125 (pMAV-*hCDKL5_5*) strain onto a TYP agar plate containing 100 µg/mL ampicillin. Incubate it at 15 °C for about 36 h. The plate can be stored up to three days at 4 °C, carefully sealed with Parafilm to avoid oxygen availability to the cells (*see* **Note 6**).

2. Pick a single colony and inoculate it into 2 mL of liquid TYP medium supplemented with 100 µg/mL ampicillin in a 15 mL snap-cap inoculation tube, and incubate at 15 °C under vigorous shaking (180 rpm) for 24 h.

3. Perform a 1:100 dilution of the culture of step 2 in 10 mL of GG medium supplemented with Schatz Salts and 100 µg/mL ampicillin in a 100 mL Erlenmeyer flask and incubate for 24 h at 15 °C under shaking (180 rpm).

4. Dilute the pre-culture of step 3 to 0.2 OD_{600nm} in 50 mL of GG medium supplemented with Schatz Salts and 25 µg/mL ampicillin in a 250 mL Erlenmeyer flask and incubate

for 48-72 h at 0 °C under shaking (160 rpm). The final biomass concentration should be about 2.5-3.0 OD_{600nm}.

3.3.2. Production process

1. Inoculate the amount of pre-culture required to obtain a starting concentration of OD_{600nm}= 0.1. To calculate it, register the optical density of the pre-culture at 600nm using a spectrophotometer. Calculate the volume of inoculum by using the following formula:

mL inoculum = (culture OD_{600nm} * mL culture volume)/ pre-culture OD_{600nm})

2. Monitor the cell growth by measuring the optical density at 600nm as described above. Register the data of at least two measurements to avoid the technical error. When the cell density reaches an OD_{600nm} of 0.8-1.2, which corresponds to the exponential phase, induce the recombinant gene expression with D-galactose addition. Add the required amount of 1 M D-galactose sterile stock solution to obtain the optimal inducer concentration of 40 mM.

3. At different times (8h, 24h and 48h) after the induction collect two samples from each culture corresponding to an OD_{600nm} of 1 and 20 OD, respectively. Calculate the volume of each sample using the following formula:

mL sample = X OD_{600nm}/ culture optical density OD_{600nm}

1 OD pellets will be used for total cellular expression analysis, while 20 OD pellets will be lysed for solubility screenings. Collect samples by centrifuging the calculated volume for 15 min at 4,000x*g* at 4°C. Discard the supernatant and store the biomass indefinitely at -80 °C.

4. Plot the optical density values versus the time of cultivation in graphs. In a typical process (Fig.1) during the first exponential phase the highest specific growth rate is reached (μ_{max} = 0.03 h^{-1}).

3.4. CDKL5 production analysis

To analyse the *h*CDLK5_5 production, a cellular lysis followed by Western blotting analysis is required.

3.4.1. Cell lysis

1.. Add 1 tab of cOmplete mini Protease Inhibitor cocktail tablet to 10 mL of Lysis buffer and mix until the tablet is completely dissolved (*see* **Note 7**).

2. Resuspend the collected bacterial pellet (OD_{600nm}= 20) at different fermentation time points in 2.0 mL of Lysis buffer by pipetting or vortexing.

3. Sonicate the samples for 15 minutes at 15% Amplitude (15 seconds pulse, 30 seconds pause) using a Bandelin Sonoplus HD 3200 sonicator with an MS72 probe or equivalent. During the sonication process, place the sample in an ice/water bath to avoid sample heating.

5. Centrifuge the suspensions at 14,000x*g* for 20 min at 4 °C. Recover the resulting supernatant containing the total soluble protein extract for further analysis. Keep the protein extract on ice or store it a 4 °C for no longer than 2h.

3.4.2. Western blot protein detection

Protein samples were separated by sodium dodecyl sulphate polyacrylamide gel electrophoresis (SDS-PAGE) (10%,w/v acrylamide) and transferred to a polyvinylidenedifluoride membrane (PVDF) with a Tans-Blot SD Semi-Dry Transfer Cell and immunodetection was performed with the primary anti-CDKL5 antibody.

1. For production analysis in total extracts, solubilize the 1 OD pellets of each sample prepared at 3.3.2 with 60 µL of 4x SDS-PAGE loading buffer and boil the samples for 20 min at 95 °C. For the solubility analysis, mix 40 μl of the soluble fractions collected as

indicated at 3.4.1 with 13 μl of 4x SDS-PAGE loading buffer and boil for 5 min at 95 °C. Load 5 μl of the samples onto 10% SDS-PAGE gels. Run the gels for 90 min at constant 100 Volts.

2. Wash the gel 3 times with transfer buffer for 10 min.

3. Transfer the proteins on a 0.2 μm PVDF membrane previously activated in methanol according to the manufacturer's instructions using a Tans-Blot SD Semi-Dry Transfer Cell for 30 min at constant 15 Volts.

4. Block the membrane for 1h at room temperature (RT) in blocking buffer under shaking.

5. Dilute anti-CDKL5 antibody 1:1,000 in blocking buffer by diluting 20 μl of antibody in 20 mL. Incubate the membrane with the primary antibody solution for 1h at RT under shaking.

6. Discard the primary antibody solution and wash the membrane 3 times with Western blot washing buffer for 10 min.

7. Dilute anti-mouse HRP-conjugated antibody 1:20,000 in blocking buffer by diluting 1 μl of antibody in 20 mL. Incubate the membrane with the primary antibody solution for 1 h at RT under shaking.

8. Discard the secondary antibody solution and wash the membrane 5 times with Western blot washing buffer for 10 minutes.

9. Develop the Western blot using the Clarity Western ECL substrate or equivalent following the manufacturer's instructions.

10. Acquire the chemiluminescent image with a ChemiDoc MP Imager and analyse with the IMAGE-lab Software.

The analysis (Fig. 2) reveals that *h*CDKL5_5 is produced in the different tested conditions and that the different liquid-to-gas ratios slightly influence the production yields, while the sampling timing after induction influences the amount of recovered protein. As shown in Fig 2B, the higher amount of protein is obtained 8 h after induction in total protein extracts. It is worth noting that the protein is present in the soluble fraction (Fig. 3) and, although a degradation pattern is present, the main product is still the larger one. In conclusion, when produced at 0 °C, the human recombinant protein CDKL5_5 resulted to be produced in soluble form. Further investigation has to be carried out to increase the production yields and to reduce proteolysis of the recombinant protein.

4. Notes

1. GelRed can be added while the gel solution is still hot.

2. The GG medium complemented with Schatz Salts can be stored one day at RT.

3. The galactose-inducible pMAV plasmid was used to produce recombinant proteins at subzero temperature (6). pMAV deriving from the pUC18 plasmid, is characterised by the presence of: i) the pJB3-derived OriT (19), a DNA fragment responsible for the initiation of the conjugative transfer between *E. coli* S17-1 λ*pir* strain (donor) and the psychrophilic cells (acceptor); ii) a pUC18-derived polylinker wherein the target gene can be cloned; iii) the *E. coli blaM* gene, encoding a mesophilic β-lactamase which is used for the selection of the recombinant clones; iv) OriC, the origin of replication allowing the plasmid to replicate in *E. coli*; v) the T/R box, a DNA fragment containing the cold-adapted origin of replication (OriR) (16); vi) the DNA region upstream to the *Phgal* operon containing both the *PhgalTK* promoter and the gene coding for *Ph*GalR (6) ;vii) the *TaspC*, the transcription termination signal of the aspartate aminotransferase gene (*aspC*) isolated from *P. haloplanktis* TAC125 (20).

4. pET28a-*hCDKL5* vector was kindly provided by Prof. E. Ciani, University of Bologna.

5. Keep bacterial cultures cold while working with or transporting them using ice during the growths at 15 °C and directly sampling in the incubator during expression experiments at 0 °C.

6. The psychrophilic bacteria are able to grow at the storage temperature of 4 °C. Limiting oxygen availability can reduce growth but not avoid it.

7. The addition of a wide spectrum protease inhibitors cocktail to the lysis buffer to prevent proteolytic degradation of the recombinant product is crucial because of the intrinsic flexibility of *h*CDKL5_5, which makes it an easy target of endogenous proteases.

5. References

1. Médigue C, Krin E, Pascal G, et al (2005) Coping with cold: The genome of the versatile marine Antarctica bacterium *Pseudoalteromonas haloplanktis* TAC125. Genome Res 15:. https://doi.org/10.1101/gr.4126905
2. Parrilli E, Tutino ML (2017) Heterologous protein expression in *Pseudoalteromonas haloplanktis* TAC125 Psychrophiles: From Biodiversity to Biotechnology: Second Edition: 513-525
3. Vigentini I, Merico A, Tutino ML, et al (2006) Optimization of recombinant human nerve growth factor production in the psychrophilic *Pseudoalteromonas haloplanktis*. J Biotechnol 127:141–150. https://doi.org/10.1016/j.jbiotec.2006.05.019
4. Corchero JL, Gasser B, Resina D, et al (2013) Unconventional microbial systems for the cost-efficient production of high-quality protein therapeutics. Biotechnol Adv 31:140–153. https://doi.org/10.1016/j.biotechadv.2012.09.001
5. Unzueta U, Vázquez F, Accardi G, et al (2015) Strategies for the production of difficult-to-express full-length eukaryotic proteins using microbial cell factories: production of human alpha-galactosidase A. Appl Microbiol Biotechnol 99:5863–5874. https://doi.org/10.1007/s00253-014-6328-9
6. Sannino F, Giuliani M, Salvatore U, et al (2017) A novel synthetic medium and expression system for subzero growth and recombinant protein production in *Pseudoalteromonas haloplanktis* TAC125. Appl Microbiol Biotechnol 101:. https://doi.org/10.1007/s00253-016-7942-5
7. Fehr S, Wilson M, Downs J, et al (2013) The CDKL5 disorder is an independent clinical entity associated with early-onset encephalopathy. Eur J Hum Genet 21:266–273. https://doi.org/10.1038/ejhg.2012.156

8. Hector RD, Kalscheuer VM, Hennig F, et al (2017) CDKL5 variants . Neurol Genet 3:e200. https://doi.org/10.1212/nxg.0000000000000200

9. Alowolodu O, Johnson G, Alashwal L, et al (2016) Intrinsic disorder in spondins and some of their interacting partners. Intrinsically Disord Proteins 4:e1255295. https://doi.org/10.1080/21690707.2016.1255295

10. Fahmi M, Yasui G, Seki K, et al (2019) In silico study of Rett syndrome treatment-related genes, MECP2, CDKL5, and FOXG1, by evolutionary classification and disordered region assessment. Int J Mol Sci 20:1–19. https://doi.org/10.3390/ijms20225593

11. Kameshita I, Sekiguchi M, Hamasaki D, et al (2008) Cyclin-dependent kinase-like 5 binds and phosphorylates DNA methyltransferase 1. Biochem Biophys Res Commun 377:1162–1167. https://doi.org/10.1016/j.bbrc.2008.10.113

12. Katayama S, Inazu T (2019) Straightforward and rapid method for detection of cyclin-dependent kinase-like 5 activity. Anal Biochem 566:58–61. https://doi.org/10.1016/j.ab.2018.11.013

13. Wilmes B, Kock H, Glagla S, et al (2011) Cytoplasmic and periplasmic proteomic signatures of exponentially growing cells of the psychrophilic bacterium *Pseudoalteromonas haloplanktis* TAC125. Appl Environ Microbiol 77:1276–1283. https://doi.org/10.1128/AEM.01750-10

14. Hector RD, Dando O, Landsberger N, et al (2016) Characterisation of CDKL5 Transcript Isoforms in Human and Mouse. PLoS One 11:1–22. https://doi.org/10.1371/journal.pone.0157758

15. Fichou Y, Nectoux J, Bahi-Buisson N, et al (2011) An isoform of the severe encephalopathy-related CDKL5 gene, including a novel exon with extremely high sequence conservation, is specifically expressed in brain. J Hum Genet

56:52–57. https://doi.org/10.1038/jhg.2010.143

16. Tutino ML, Duilio A, Parrilli E, et al (2001) A novel replication element from an Antarctic plasmid as a tool for the expression of proteins at low temperature. Extremophiles 5:. https://doi.org/10.1007/s007920100203

17. Lessard JC (2013) Molecular cloning. Methods Enzymol 529: 85-98. https://doi.org/10.1016/B978-0-12-418687-3.00007-0

18. Parrilli E, Duilio A, Tutino ML (2008) Heterologous protein expression in psychrophilic hosts. Psychrophiles: From Biodiversity to Biotechnology: 365-379

19. Blatny JM, Brautaset T, Winther-Larsen HC, et al (1997) Construction and use of a versatile set of broad-host-range cloning and expression vectors based on the RK2 replicon. Appl Environ Microbiol 63:370–379. https://doi.org/10.1128/aem.63.2.370-379.1997

20. Birolo L, Tutino ML, Fontanella B, et al (2000) Aspartate aminotransferase from the Antarctic bacterium *Pseudoalteromonas haloplanktis* TAC 125. Cloning, expression, properties, and molecular modelling. Eur J Biochem 267:2790-802. doi: 10.1046/j.1432-1327.2000.01299.x

7. Figure captions

Figure 1: Growth profiles of recombinant *P. haloplanktis* TAC125 (pMAV-*hCDKL5_5*) at 0°G in GG synthetic medium performed at different percentage of liquid gas ratio.

Figure 2: *h*CDKL5_5 production in *P. haloplanktis* TAC125 (pMAV-*hCDKL5_5*). **A**) SDS-PAGE analysis of total lysates of *P. haloplanktis*TAC125 (pMAV-*hCDKL5_5*)

grown at 0 °C in GG. **B**) Analysis of *h*CDKL5_5 recombinant production by Western blot using an anti-CDKL5 monoclonal antibody.

Figure 3: Western Blotting analysis of the soluble fraction of *P.haloplanktis* TAC125 (pMAV-*hCDKL5_5*) cell lysate 8h after induction with 40 mM galactose at 0°C. **A**) 5 µg of soluble fraction. **B**) 10 µg of soluble fraction.

Acknowledgements

This work was supported by Programma Nazionale di Ricerca in Antartide Project PNRA18_00335, and by the Italian parent's association "La fabbrica dei sogni 2- New developments for Rett syndrome".

Fig 1

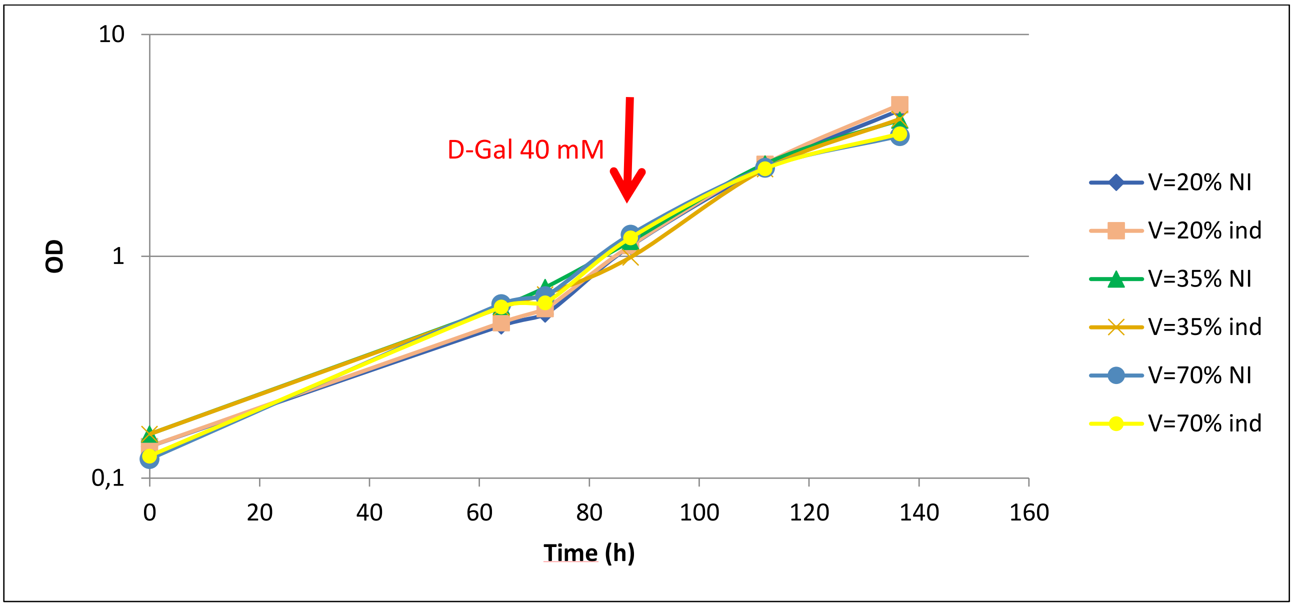
10
1
0,1
OD
D-Gal 40 mM
0
20
40
60
80
100
120
140
160
Time (h)
V=20% NI
V=20% ind
V=35% NI
V=35% ind
V=70% NI
V=70% ind

Fig 2

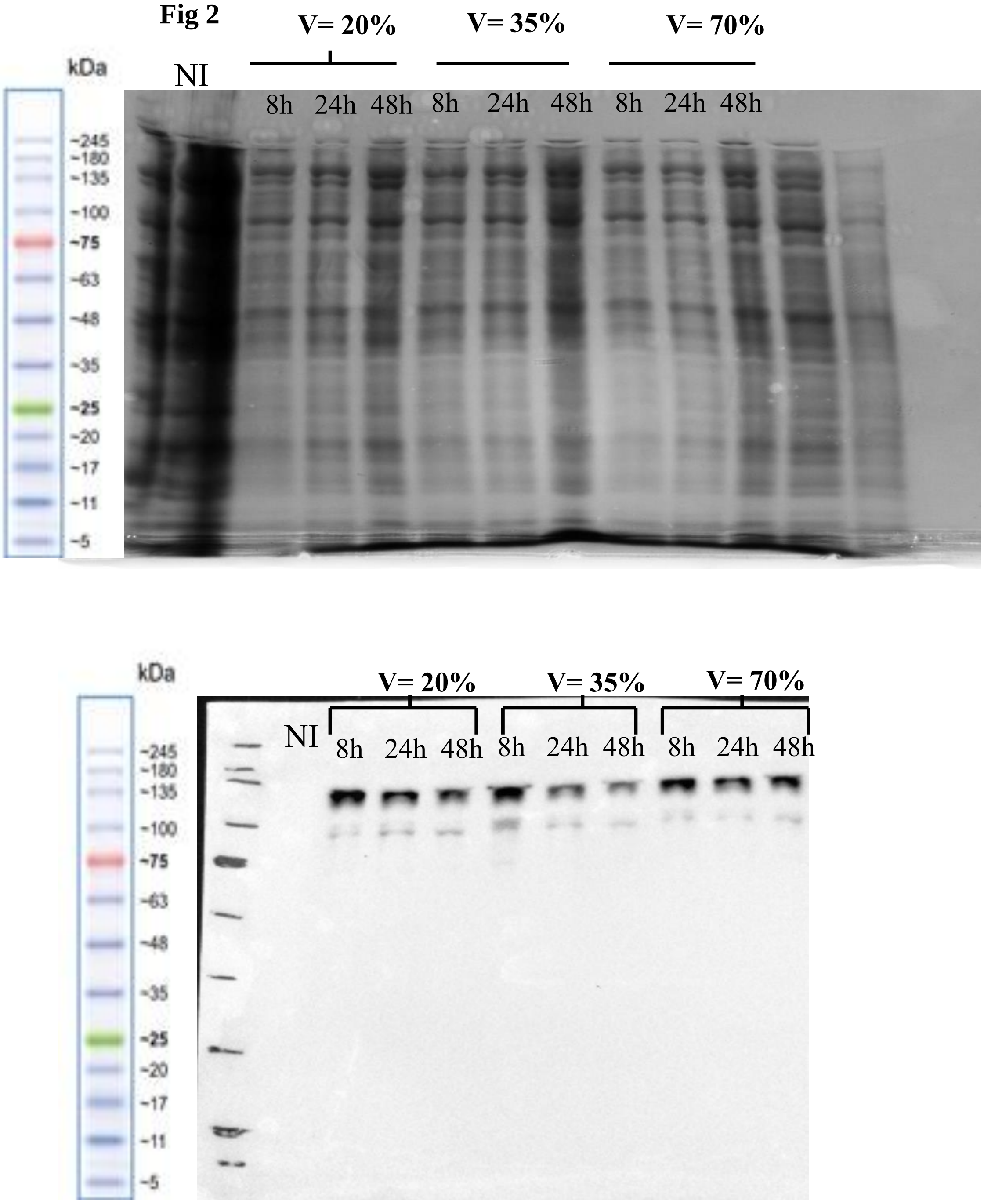
V= 20%
V= 35%
V= 70%
kDa
NI
8h 24h 48h 8h 24h 48h 8h 24h 48h
~245
~180
~135
~100
~75
~63
~48
~35
~25
~20
~17
~11
~5
kDa
V= 20%
V= 35%
V= 70%
NI
8h 24h 48h 8h 24h 48h 8h 24h 48h
~245
~180
~135
~100
~75
~63
~48
~35
~25
~20
~17
~11
~5

Fig 3

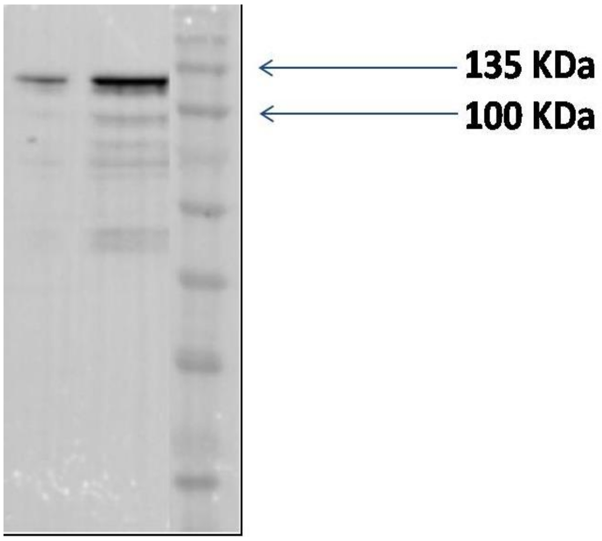

Supplement: Supplementary file 1 [file metabolites-11-00491-s001.zip › Chapter 14_ Tutino_REVISED 04_11_2020 final.pdf]
