# Supplementary material for: Modelling hCDKL5 Heterologous Expression in Bacteria
Source: Metabolites. 2021 Jul 28;11(8):491. doi: 10.3390/metabo11080491 (PMC8401935; doi:10.3390/metabo11080491)
Supplement: Supplementary file 1 [file metabolites-11-00491-s001.zip › Supplementary_information_S1_CDKL5.pdf]

Supplementary information of: **Modelling hCDKL5 heterologous expression in bacteria**

Marco Fondi[1,2*], Stefano Gonzi[1], Mikolaj Dziurzynski[3], Paola Turano[4], Veronica Ghini[4], Marzia Calvanese[5], Andrea Colarusso[5], Concetta Lauro[5], Ermenegilda Parrilli[5], Maria Luisa Tutino[5]

[1] Department of Biology, University of Florence, Italy
[2] Centro Studi Dinamiche Complesse (CSDC), University of Florence, Italy
[3] Department of Environmental Microbiology and Biotechnology, Institute of Microbiology, Faculty of Biology, University of Warsaw, Poland
[4] Center of Magnetic Resonance (CERM), University of Florence, Italy
[5] Dipartimento di Scienze Chimiche, Complesso Universitario Monte Sant'Angelo, Napoli, Italy.

* Corresponding author: marco.fondi@unifi.it

Comparison of predicted growth rates with different versions of PhTAC125 genome-scale reconstruction

In order to evaluate whether the changes made to the original formulation of the model, we repeated the simulations reported in Fondi et al. 2014. Results shown below, show that the model is still in good agreement with available experimental data on PhTAC125 growth on minimal media.

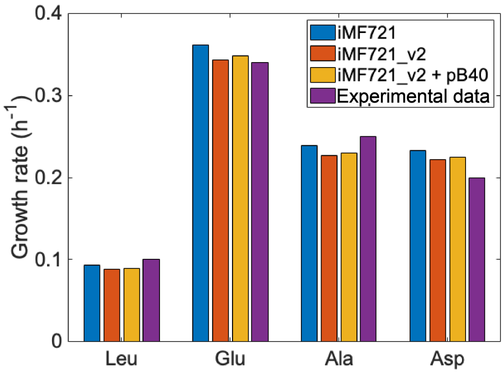

Figure S1. Comparison among predicted growth rates of different versions of PhTAC125 metabolic reconstructions. iMF721 refers to the original version of the model as presented in Fondi et al. 2014. iMF721_v2 to the updated version of the model as described in the main text. iMF721_v2 + pB40 refers to this latter model, plus the pB40 plasmid used for CDKL5 protein production.

Growth and CDKL5 production values

Table S1. WT and CDKL5 strains growth parameters over an 8 h growth period.

	Final OD (8h)	m (h^{-1})
WT (wt)	3.47	0.169
CDKL5 (recomb)	2.55	0.124

Glutamate and gluconate uptake experimental data

To establish the consumption rate of the carbon sources included in the medium, *Ph*TAC125 bacterial cells were grown GG medium containing 5 g/L of L-glutamic acid monosodium salt monohydrate and 5 g/L D-gluconic acid sodium salt in a Stirred Tank Reactor 3 L fermenter in the conditions described in the main text (Material and Methods). After the growth, the supernatants were recovered, filtered through membranes with a pore diameter of 0.22 µm, and stored at -80 °C before undergoing glutamate and gluconate quantification using NMR as described in the main text. Results of this experiment are reported in Figure S2 together with OD.

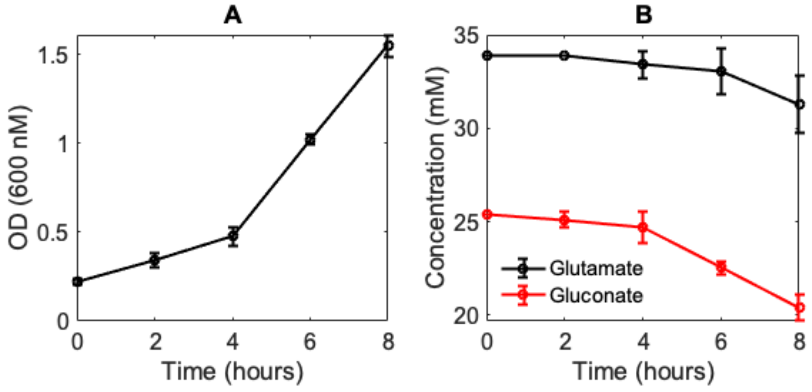

Figure S2. Growth (**A**) and glutamate and gluconate consumption (**B**) of PhTAC125 cells over an 8 hours period.

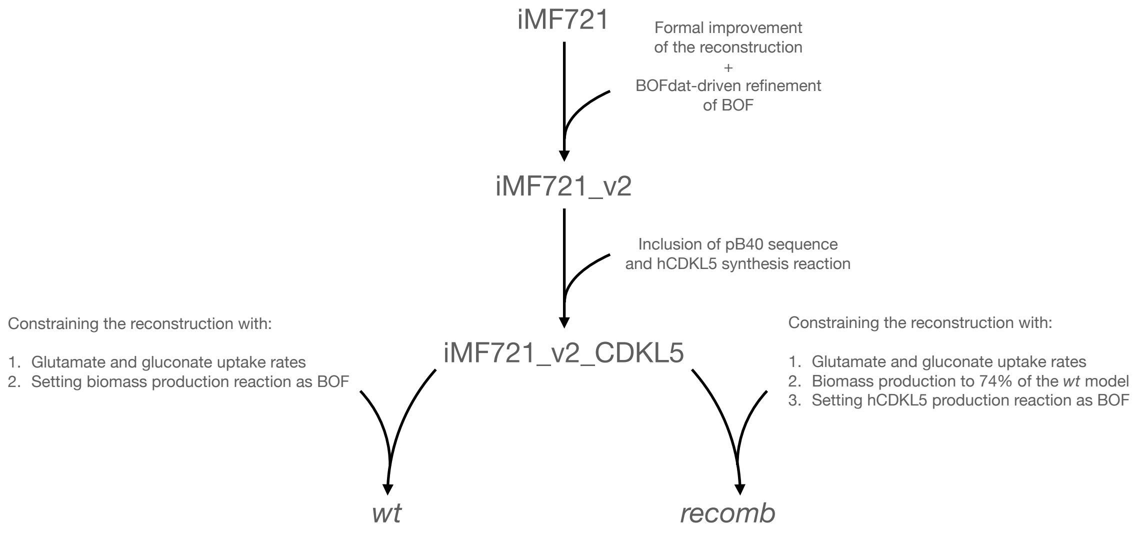

Figure S3. The different steps performed in this work to obtain the *wt* and *recomb* metabolic reconstructions.

Table S2. Glutamate and gluconate consumption parameters during growth in GG medium with a initial volume of 1.6 L.

	MW (g/mol)	Initial concentration (g/L)	Final concentration (mM)	Initial concentration (mM)	Yield	Uptake rates (mmol/g DCW h^{-1})	
Glutamate	147.13	5	31.3	33.9	0.70	0.3463	
Gluconate	196.16	5	20.4	25.4	0.36	0.6660	

Table S3. The complete list of target reactions identified through FSEOF.

**Slope**	**rowID**	**Enzyme ID**	**Enzyme Name**	**Direction**	**Gr Rule**
4.574	7	rxn00802	N-(L-Argininosuccinate) arginie-lyase	1	PSHA_RS11280
1.039	15	rxn00011	pyruvatethiamin diphosphate acetaldehydetransferase	0	((PSHA_RS13615 and PSHA_RS13620 and PSHA_RS01930) or (PSHA_RS17075 and PSHA_RS17070))
49.424	22	rxn05466	Ammonia transport via diffusion	1	PSHA_RS11135
2.416	30	rxn02476	Phosphoenolpyruvate3-phosphoshikimate	1	PSHA_RS07005
1235,34	45	rxn01985	Deoxyinosineorthophosphate ribosyltransferase	-1	PSHA_RS15265
6.436	54	rxn02186	2,3-Dihydroxy-3-methylbutanoateNADP+ oxidoreductase (isomerizing)	1	PSHA_RS04145
2.416	57	rxn02212	2-Dehydro-3-deoxy-D-arabino-heptonate 7-phosphate phosphate-lyase	0	PSHA_RS13355
3.899	63	rxn02160	L-Histidinol-phosphate phosphohydrolase	0	PSHA_RS17195
5.707	83	rxn02789	2-Isopropylmalate hydro-lyase	1	(PSHA_RS14190 and PSHA_RS14195)
5.707	107	rxn00902	3-Carboxy-3-hydroxy-4-methylpentanoate 3-methyl-2-oxobutanoate-lyase	-1	PSHA_RS14205
5.535	111	rxn00503	L-1-Pyrroline-5-carboxylateNAD+ oxidoreductase	-1	PSHA_RS11170
3.899	129	rxn00863	L-HistidinalNAD+ oxidoreductase	1	PSHA_RS17205
7.467	148	rxn00423	serine O-acetyltransferase	-1	PSHA_RS03150
1.039	150	rxn03436	(S)-2-Aceto-2-hydroxybutanoateNADP+ oxidoreductase (isomerizing)	1	PSHA_RS04145
3.899	173	rxn02159	L-HistidinolNAD+ oxidoreductase	1	PSHA_RS17205
7.467	182	rxn00649	O3-Acetyl-L-serine acetate-lyase (adding hydrogen sulfide)	-1	PSHA_RS09475

2.416	252	rxn01332	PhosphoenolpyruvateD-erythrose-4-phosphate	0	PSHA_RS17530
7.461	253	rxn00566	L-Cysteine L-homocysteine-lyase (deaminating)	-1	PSHA_RS09510
1235,34	254	rxn01858	Deoxyadenosine aminohydrolase	-1	PSHA_RS00520
3.899	295	rxn02834	Phosphoribosyl-ATP pyrophosphohydrolase	0	PSHA_RS17160
3.899	302	rxn03175	N-(5'-Phospho-D-ribosylformimino)-5-amino-1-	1	PSHA_RS17185
3.899	304	rxn02473	D-erythro-1-(Imidazol-4-yl)glycerol 3-phosphate hydro-lyase	0	PSHA_RS17195
4.574	317	rxn01637	N2-Acetyl-L-ornithine2-oxoglutarate aminotransferase	-1	(PSHA_RS00975 or PSHA_RS16905)
5.545	329	rxn00313	meso-2,6-Diaminoheptanedioate carboxy-lyase	0	(PSHA_RS00475 or PSHA_RS00895)
10.441	376	rxn00285	SuccinateCoA ligase (ADP-forming)	0	(PSHA_RS08065 and PSHA_RS08060)
4.574	395	rxn00192	Acetyl-CoAL-glutamate N-acetyltransferase	0	PSHA_RS11280
6.436	420	rxn00898	2,3-Dihydroxy-3-methylbutanoate hydro-lyase	0	(PSHA_RS13610 or PSHA_RS15485)
2.416	421	rxn01739	ATPshikimate 3-phosphotransferase	0	PSHA_RS13360
301	445	rxn00414	Carbon-dioxideL-glutamine amido-ligase (ADP-forming,	0	(PSHA_RS06060 and PSHA_RS06065)
16.403	448	rxn00260	L-Aspartate2-oxoglutarate aminotransferase	-1	(PSHA_RS06515 or PSHA_RS04660 or PSHA_RS00305)
74.285	449	rxn05937	FerredoxinNADP+ oxidoreductase	1	PSHA_RS15270
664.470	450	rxn05319	H2Ot5	-1	SPONTANEOUS
1.770	456	rxn00527	L-Tyrosine2-oxoglutarate aminotransferase	-1	PSHA_RS17200
5.194	471	rxn01974	LL-2,6-Diaminoheptanedioate 2-epimerase	1	PSHA_RS00470
5.194	473	rxn01644	L-Aspartate-4-semialdehyde hydro-lyase (adding pyruvate and	0	(PSHA_RS16725 or PSHA_RS00905)
4.574	506	rxn02465	N-Acetyl-L-glutamate-5-semialdehydeNADP+ 5-oxidoreductase	-1	PSHA_RS11300
37.143	519	rxn12822	L-Glutamateferredoxin oxidoreductase (transaminating)	1	PSHA_RS14360
1.039	522	rxn03437	(R)-2,3-Dihydroxy-3-methylpentanoate hydro-lyase	0	(PSHA_RS13610 or PSHA_RS15485)
3.899	525	rxn02320	5-Amino-2-oxopentanoate2-oxoglutarate aminotransferase	-1	PSHA_RS17200
2.416	534	rxn01740	ShikimateNADP+ 5-oxidoreductase	-1	PSHA_RS00155
460	576	rxn05651	sulfate transport in via proton symport	1	PSHA_RS06900
4.574	584	rxn01434	L-CitrullineL-aspartate ligase (AMP-forming)	1	PSHA_RS11285

466	586	rxn00693	5-MethyltetrahydrofolateL-homocysteine S-methyltransferase	1	(PSHA_RS10965 and PSHA_RS10970)
15.768	591	rxn00337	ATPL-aspartate 4-phosphotransferase	0	(PSHA_RS13395 or PSHA_RS06160 or PSHA_RS02605 or PSHA_RS11720)
2.416	616	rxn02213	3-Dehydroquinate hydro-lyase	1	PSHA_RS01310
5.194	634	rxn01973	N-Succinyl-LL-2,6-diaminoheptanedioate amidohydrolase	1	PSHA_RS06550
460	686	rxn00623	hydrogen-sulfideNADP+ oxidoreductase	-1	(PSHA_RS00790 and PSHA_RS00785)
21.970	697	rxn01477	6-Phospho-D-gluconate hydro-lyase	0	PSHA_RS06715
5.194	706	rxn03031	Succinyl-CoA2,3,4,5-tetrahydropyridine-2,6-dicarboxylate	1	(PSHA_RS10075 or PSHA_RS00900)
1.039	716	rxn03435	(R)-2,3-Dihydroxy-3-methylpentanoateNADP+ oxidoreductase	-1	PSHA_RS04145
1.334	757	rxn00493	L-Phenylalanine2-oxoglutarate aminotransferase	-1	PSHA_RS17200
5.707	759	rxn01208	2-Oxo-4-methyl-3-carboxypentanoate decarboxylation	0	SPONTANEOUS
3.899	766	rxn00789	1-(5-Phospho-D-ribosyl)-ATPpyrophosphate phosphoribosyl-transferase	-1	PSHA_RS17210
460	783	rxn05256	APSPTi	0	PSHA_RS00795
1.334	807	rxn01000	Prephenate hydro-lyase (decarboxylating)	0	PSHA_RS04630
15.768	816	rxn01643	L-Aspartate-4-semialdehydeNADP+ oxidoreductase (phosphorylating)	-1	(PSHA_RS10285 or PSHA_RS17375)
5.194	832	rxn02929	2,3,4,5-TetrahydrodipicolinateNADP+ oxidoreductase	-1	PSHA_RS06055
5.707	854	rxn02811	3-Isopropylmalate hydro-lyase	-1	(PSHA_RS14190 and PSHA_RS14195)
4.574	861	rxn01019	Carbamoyl-phosphateL-ornithine carbamoyltransferase	1	PSHA_RS11290
3.899	871	rxn03135	Imidazole-glycerol-3-phosphate synthase	0	(PSHA_RS17190 and PSHA_RS17165)
4.574	872	rxn00469	N2-Acetyl-L-ornithine amidohydrolase	1	PSHA_RS11305
5.194	900	rxn03087	N-Succinyl-L-2,6-diaminoheptanedioate2-ocoglytarate	-1	(PSHA_RS00975 or PSHA_RS16905)
3.899	904	rxn02835	1-(5-phospho-D-ribosyl)-AMP 1,6-hydrolase	1	PSHA_RS17160
4.574	923	rxn01917	ATPN-acetyl-L-glutamate 5-phosphotransferase	1	PSHA_RS11295
21.970	927	rxn03884	2-dehydro-3-deoxy-D-gluconate-6-phosphate	1	PSHA_RS06720

1235,34	946	rxn00836	IMPpyrophosphate phosphoribosyltransferase	1	PSHA_RS02990
2.416	952	rxn01255	5-O-(1-Carboxyvinyl)-3-phosphoshikimate phosphate-lyase	0	PSHA_RS04795
1.039	953	rxn03194	(S)-2-Aceto-2-hydroxybutanoate pyruvate-lyase (carboxylating)	1	((PSHA_RS13615 and PSHA_RS13620) or (PSHA_RS17075 and PSHA_RS17070))
21.970	1017	rxn01476	6-Phospho-D-glucono-1,5-lactone lactonohydrolase	1	PSHA_RS05660
216.684	1021	rxn05312	PIt6	1	PSHA_RS01555
1.770	1025	rxn01268	PrephenateNAD+ oxidoreductase(decarboxylating)	0	PSHA_RS04625
460	1032	rxn09240	Sulfate adenyltransferase	1	(PSHA_RS01040 and PSHA_RS01035)
5.707	1084	rxn03062	3-IsopropylmalateNAD+ oxidoreductase	1	AUTOCOMPLETION
3.104	1086	rxn01256	Chorismate pyruvatemutase	1	AUTOCOMPLETION
538.953	1099	EX_cpd00067_e	EX_H+_e	-1	
664.470	1113	EX_cpd00001_e	EX_H2O_e	0	
460	1130	EX_cpd00048_e	EX_Sulfate_e	-1	
216.684	1140	EX_cpd00009_e	EX_Phosphate_e	-1	
49.424	1142	EX_cpd00013_e	EX_NH3_e	-1	
6.436	1267	rxn00003	2-Acetolactate pyruvate-lyase (carboxylating)	-1	(PSHA_RS17075 and PSHA_RS17070)
100	1328	CDKL5,c	CDKL5,c	0	
1	1329	EX_cdkl5[c]	EX_cdkl5[c]	0	
